# A New Antigen Retrieval Technique for Human Brain Tissue

**DOI:** 10.1371/journal.pone.0003378

**Published:** 2008-10-13

**Authors:** Raúl Alelú-Paz, Ignacio Iturrieta-Zuazo, William Byne, Vahram Haroutunian, Mercedes García-Villanueva, Alberto Rábano, María García-Amado, Lucía Prensa, José Manuel Giménez-Amaya

**Affiliations:** 1 Departamento de Anatomía, Histología y Neurociencia, Facultad de Medicina, Universidad Autónoma de Madrid, Madrid, Spain; 2 Laboratorio de Neuromorfología Funcional, Clínica Universitaria, Universidad de Navarra, Pamplona, Spain; 3 Department of Psychiatry, Mount Sinai School of Medicine, New York, New York, United States of America; 4 Bronx Veterans Affairs Medical Center, Bronx, New York, United States of America; 5 Departamento de Anatomía Patológica, Hospital Ramón y Cajal, Madrid, Spain; 6 Laboratorio de Neuropatología, Hospital de Alcorcón, Madrid, Spain; University of Auckland, New Zealand

## Abstract

Immunohistochemical staining of tissues is a powerful tool used to delineate the presence or absence of an antigen. During the last 30 years, antigen visualization in human brain tissue has been significantly limited by the masking effect of fixatives. In the present study, we have used a new method for antigen retrieval in formalin-fixed human brain tissue and examined the effectiveness of this protocol to reveal masked antigens in tissues with both short and long formalin fixation times. This new method, which is based on the use of citraconic acid, has not been previously utilized in brain tissue although it has been employed in various other tissues such as tonsil, ovary, skin, lymph node, stomach, breast, colon, lung and thymus. Thus, we reported here a novel method to carry out immunohistochemical studies in free-floating human brain sections. Since fixation of brain tissue specimens in formaldehyde is a commonly method used in brain banks, this new antigen retrieval method could facilitate immunohistochemical studies of brains with prolonged formalin fixation times.

## Introduction

Immunohistochemical staining of tissues is a powerful tool used to delineate the presence or absence of an antigen. During the last 30 years, antigen visualization in the human brain tissue has been significantly limited by the masking effect of fixatives such as formaldehyde, which is widely used and prepared from a commercial concentrated formalin (40% solution of formaldehyde) that is diluted to a 10% solution (10% formalin) [1]. Briefly, the process of fixation implies the denaturalization of a biological molecule, specifically changing its shape, which enables the molecule to withstand the rigors of the tissue processing by locking the secondary structure [2] and preventing the degradation of this molecule by way of endogenous or microbial enzymes [3].

Formalin-fixed tissue is routinely used in pathology specimens and gives excellent morphology. Therefore, such tissue is preferred for immunohistochemical staining. The antigen masking effect of the formalin fixation process has required the use of antigen retrieval protocols before immunohistochemical staining. Since the early 1990s, several procedures for antigen retrieval (AR) have been developed and proven to be effective for immunohistochemistry on light microscopic preparations in human brain tissue [4]. These techniques are based on the immersion of the sections in various solutions with different pH and at high temperatures for variable times, in order to expose the highest number of antigenic epitopes [5]. The effect of heating is probably the single most important factor for AR [1], [6]–[9], although other factors, such as the pH of the solutions, are also important [10], [11].

A commonly used technique for AR in brain tissue from various animal sources before immunohistochemical or histochemical staining is the heating in citrate buffer, pH 6.0 for times that range from 20 to 40 minutes [12]. This procedure has been demonstrated to be valid for AR in human brains fixed in 4% paraformaldehyde for a short period of time [13]. However, it does not work well enough in tissue fixed and stored for long periods in formaldehyde. It is known that the duration of formalin fixation is crucial to the retention of antigen expression [14] but, unfortunately, fixation time is not closely controlled routinely and sections are often fixed for much longer times than desired. Improved methods for AR in tissues subjected to prolonged fixation in formalin are, therefore, needed for optimal immunohistochemical and histochemical staining.

In the present study, we describe a new method for AR in formalin-fixed human brain tissue and examined the effectiveness of this protocol to reveal masked antigens in tissues with both short and long formalin fixation times. This new method, which is based on citraconic acid, has not been previously used in brain tissue although it has been employed in various other tissues such as tonsil, ovary, skin, lymph node, stomach, breast, colon, lung and thymus.

## Methods

In developing this new AR method, we have used seven human brains from individuals of both sexes (two males and five females), fixed and stored in formaldehyde for variable periods of time, ranging from 10 days to 7 years (Table 1). Four human brains were provided by the Brain Bank of the Neuropathology Laboratory of the Hospital de Alcorcón (Madrid, Spain) and the Alzheimer's disease and Schizophrenia Brain Bank of the Mount Sinai Hospital (New York City, USA), with the corresponding written consents given by the patients or their relatives. Three human brains were provided by the Department of Pathology of the Hospital Ramón y Cajal (Madrid, Spain); at the time of the decease, the relatives of these patients were asked for authorization to perform the medical autopsy. Then, many medical samples were anonymized and kept in the hospital for research purposes. The biological samples of the present study were provided by these Departments after the approval of our specific project by the corresponding Ethical Committees of the hospitals where the samples were taken (Hospital de Alcorcón, Hospital Ramón y Cajal and Mount Sinai Hospital) and the Universidad Autónoma de Madrid.

**Table 1:** Data of the human specimen used in this study.

Case	Sex	Age (years)	Hemisphere	Postmortem delay (hours)	Storage time in formalin (10%)	Weight (g)	Cause of death
1	Male	72	Right	Unknown	3 years	1215	Sepsis
2	Female	66	Right	Unknown	4 years	1131	Myocardial infarct
3	Male	57	Left	3	17 days	1390	Hemorrhage
4	Female	80	Unknown	6.8	7 years	1049	Acute coronary thrombosis
5	Female	90	Unknown	7.7	7 years	1008	Septic shock
6	Female	Unknown	Left	Unknown	6 months	1350	Unkown
7	Female	20	Left	2	10 days	1100	Myocardial infarct

The slabs received were cut on a freezing microtome into 50-µm-thick coronal sections. All sections were collected with a paintbrush moistened in 0.01 M phosphate buffer (PB), pH 7.4, and stored in a cryoprotectant solution containing ethylene glycol (30%), and glycerol (30%) in 0.05 M PB. Series of free-floating sections were then immunohistochemically processed to visualize calbindin D-28K (CB), substance P (SP), glutamic acid decarboxilase (GAD 65&67), tyrosine hydroxylase (TH) and enkephalin (ENK). All stained sections were mounted on gelatine-coated slides, dehydrated, cleared in xylene, and coverslipped with Depex.

### Immunohistochemistry

Briefly, after a 0.1 M PB and 0.2% Triton X-100 rinse cycle of three ten minutes rinses, the slices were immersed in a Petri dishes (60 mm×15 mm) sealed with Parafilm® and shaken for 45 minutes in a 0.05% citraconic anhydride solution (volume for each Petri dish: 3 ml; pH 7.4) at 95°C (ImmunoSaver Antigen Retriever, Electron Microscopic Sciences, Hatfield, PA) in a water bath with shaking device (Memmert, Schwabach, Germany). In order to maintain the same conditions for all the slices used in the experiment, the tissue does not be immersed in the citraconic anhydride solution until the water bath reach the 95°C. The sections went through the PB and Triton X-100 rinse cycle and were then placed in a 50% ethanol (1∶3) and 3% H_2_O_2_ (2∶3) solution for 30 minutes to inactivate endogenous peroxidase activity. The sections went through a second PB and Triton X-100 rinse cycle and were then preincubated for 90 minutes in a solution containing 0.1 M PB and 0.5% Triton X-100, and 4% of the appropriate normal serum. Then, the sections were incubated in a solution containing 0.1 M PB and 0.5% Tritón X-100, the primary antibody, and 4% of the appropriate normal serum (Table 2). After another PB and Triton X-100 rinse cycle they were incubated for 90 minutes at room temperature in a solution containing 0.1 M PB and 0.3% Tritón X-100, the secondary antibody (0.4%), which was always biotinylated IgG (Vector Laboratories, Burlingame, CA), and with 2% of the appropriate normal serum. The slices were again rinsed in the PB and Triton X-100 rinse cycle before standard avidin-biotin complex methods (ABC Standard Kit, Vector Laboratories) were applied with 3,3-diaminobenzidine tetrahydrochloride (DAB; Sigma, St. Louis, MO) and, in some cases (GAD 65&67; CB D-28K and TH) the reaction was intensified with Nickel Sulfate Hexahydrate (Sigma, St. Louis, MO). Appropriate controls were also carried out in our case material for the immunohistochemistry technique described here; in all cases the first antibody was omitted in the protocol.

**Table 2:** Antibodies used in this study.

Primary antibody	Laboratory*	Animal source	Dilution	Time of incubation	Time of DAB
CB	Sigma	Mouse	1∶2500	Overnight / 4°C	2 min
SP	Medicorp	Rat	1∶50	Overnight / 4°C	4 min
GAD	Millipore	Rabbit	1∶1000	Overnight / 4°C	3 min
TH	Diasorin	Mouse	1∶250	Overnight / 4°C	4 min
ENK	Medicorp	Mouse	1∶50	48 h / 4°C	5 min

* Sigma: St.Louis, MO, USA; Medicorp: Montreal, Canada; Millipore: Billerica, MA, USA; Diasorin Inc: Stillwater, MN, USA.

Abbreviations: CB, calbindin-D28k; DAB, 3,3′-diaminobenzidine tetrahydrochloride; ENK, enkephalin; GAD, glutamic acid decarboxilase; SP, substance P; TH, tyrosine hydroxylase.

To determinate the optimal conditions of citraconic anhydridés protocol (heating time, temperature and heat source), we first performed a preliminary study in which different heating times (ranging from 30 minutes to 60 minutes) as well as temperatures (ranging from 85°C to 95°C) and heat sources (water bath *vs* microwave) were used in order to obtain the optimal procedure to carry out immunohistochemical stainings in formaldehyde fixed human brains. Once the optimal conditions to carry out immunohistochemical procedures were established (45 minutes in a 0.05% citraconic anhydride solution, pH 7.4, at 95°C), formalin fixed brain tissue sections were subjected to the AR method, obtained a similar intensity of immunostaining compared to a protocol widely used in immunohistochemical studies [15]. In the last protocol, it is not necessary to carry out any AR methods, because the 0.5-cm-thick brain slabs were fixed in a solution containing 4% paraformaldehyde in 0.125 M phosphate buffer, pH 7.4 during a period of 10 days, then immersed in 15% sucrose in PB at 4°C for 4–7 days in order to protect them before cutting. The sucrose solution was renewed every 2 days. For long term storage, the material was stored in PB with 15% sucrose and 0.1% sodium azide at 4°C. From here on, the protocol employed was the same as the one used in the formaldehyde slabs.

### Sources of the antibodies

Table 2 summarizes the information on the antibodies used in this study. The CB antibody was highly specific mouse monoclonal (clon 300) [16], [17]. The SP antibody was a rat monoclonal antibody that does not cross-react with other known mammalian brain peptides [18]. The GAD antibody reacts specifically with 65 and 67 isoforms. TH antibody is a mouse monoclonal antibody isolated and purified from rat PC12 cells. The monoclonal antibody used to visualize ENK does not distinguish between met-enkephalin and leu-enkephalin [19].

SP is an undecapeptide widely distributed in the central nervous system. It mediates its biological actions through the G-protein coupled to the tachykinin receptor NK1 [15], [20]. This neuropeptide plays a major role in arthritis, emesis, apoptosis, pain, gastrointestinal and respiratory function and depressive and anxiety disorders [15], [20]–[22].

GAD is the only enzyme responsible for the irreversible conversion of L-glutamic acid to GABA. There have been identified two isoforms of GAD, designated GAD_65_ and GAD_67_, accordingly with their molecular weights. As it is know, gamma-aminobutyric acid (GABA) is the principal inhibitory neurotransmitter in the mammalian central nervous system.

Regarding the TH, this enzyme converts L-tyrosine to 3,4-dihydroxyphenylalanine (DOPA), in a pathway of catecholamines. TH is present in adrenal medulla, brain and all sympathetically innervated tissues.

Finally, ENK, is an endogenous opioid and an agonist at the m-opioid receptor, involved in regulation of emotional memory, thermal pain perception, stress-induced analgesia, saliency, suppression of the affective qualities of a pain stressor and in the regulation of mood and behaviour [15], [23]–[29].

### Illustration of the results and data analysis

The experimental material of this study was analyzed using the Mai et al. atlas of the human brain (1997; 2002). The stained sections were first examined with a Nikon Eclipse 80i microscope (Nikon, Melville, NY) at 20× magnification. Microphotographs were taken with a Nikon DXM1200F digital microscope camera at 300 ppi or higher resolution and saved in TIFF format. Photographic brightness and contrast were adjusted with Adobe Photoshop (Adobe Systems, San Jose, CA) and Canvas (Deneba Systems Inc., Miami, FL).

### Nomenclature

In the thalamus, the boundaries of the nuclei in the present study were delineated according to the nomenclature suggested by Hirai and Jones [30] and Jones [31], which is derived from the nomenclature of the thalamus in non-human primates [32], [33].

### Evaluation of immunostainings

Evaluation of immunohistochemical staining for the different antibodies was done independently by four observers experienced in immunohistochemical assessment in human brains (IIZ, MGA, LPS and RAP). The intensity of immunostaining was judged as weak, moderate or strong (fibers, soma and background). Only the evaluations that were in agreement in three or more observers were considered as final.

## Results

To date, our study is the first to describe a protocol for AR that allows optimal immunohistochemical staining for several antibodies in the human brain tissue that have been fixed and stored in formaldehyde for long periods. This AR method, which utilizes a citraconic anhydride solution, has previously been shown to be superior to conventional AR methods in various tissues (Citrate buffer and Tris-HCL+5% urea) [34].

**Figure 1:**
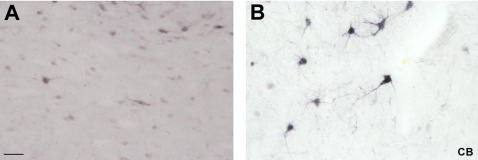
A, Immunohistochemistry for CB in the MD. High power view of some CB-ir neurons in the human mediodorsal thalamic nucleus without the employ of citraconic anhydride solution. B, high power view of CB-ir neurons in the same nucleus employing the citraconic anhydride solution. Note the enhancement in the intensity of CB-ir somata. Scale bar: 50 µm.

The immunostaining for CB in the mediodorsal thalamic nucleus in the formaldehyde fixed brains showed less intense staining of the neuropil than in the conventional immunostaining method (Figure 1). We have found in the formaldehyde fixed slices an enhancement in the intensity of CB-immunoreactive (-ir) somata. With both methods, numerous short fibers without varicosities were observed.

**Figure 2:**
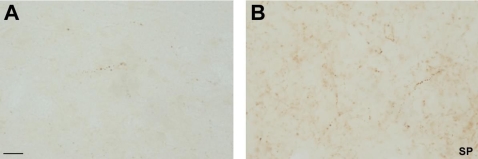
A, Neuropeptide immunohistochemistry in the human thalamus. Bright field photomicrographs in high magnification of SP fibers without citraconic anhydride solution in the anteroventral thalamic nucleus. B, bright field photomicrographs in high magnification of SP fibers with citraconic anhydride solution in the same nucleus. Note the enhancement in the intensity of SP-ir fibers and in the number of fibers. Scale bar: 25 µm.

The anteroventral thalamic nucleus in the formaldehyde fixed brains and in the conventional immunostaining method showed many intense SP-ir fibers in a weakly stained neuropil. These fibers present numerous round varicosities resembling button like structures, but no SP-ir somata were seen (Figure 2).

**Figure 3 pone-0003378-g003:**
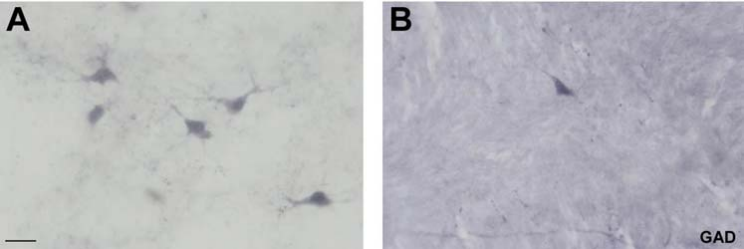
A, Interneuron immunohistochemistry in the MD. High power view of some GAD-ir neurons in the mediodorsal thalamic nucleus without the use of citraconic anhydride solution. B, high power view of a GAD-ir neuron in the mediodorsal thalamic nucleus utilizing the citraconic anhydride solution. Scale bar: 25 µm.

The immunostaining for GAD 65&67 in the mediodorsal thalamic nucleus in formaldehyde fixed brains showed a similar staining intensity that the conventional immunostaining method (Figure 3). We found weakly stained somas and intense fibers in a moderately stained neuropil. There was no apparent effect on morphology in the GAD-ir neurons, although in fomaldehyde fixed sections it is difficult to distinguish processes. The distribution of cells followed a heterogeneous pattern throughout its entire extent, as it was also seen in tissue processed by the conventional immunostaining method.

**Figure 4 pone-0003378-g004:**
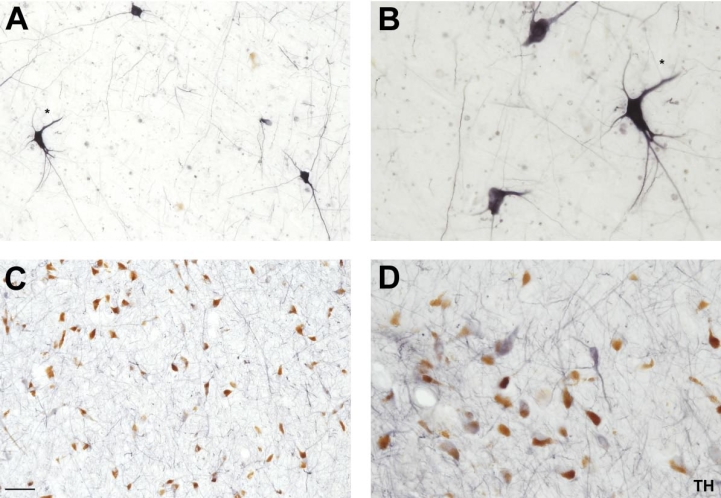
A–B, Immunohistochemistry for TH in the substantia nigra. High power view (10× and 20× magnification respectively) of TH-ir neurons in the substantia nigra employing the citraconic anhydride solution. Note the enhancement in the intensity and morphology (somas more preserved and processes more delineated) compared to the conventional immunostaining method (C–D). Scale bar: A and C 100 µm; B and D 50 µm.

The immunostaining for TH in the substantia nigra in the formaldehyde fixed brains showed an enhancement in the intensity of staining compared to the conventional immunostaining method (Figure 4), with an apparent effect on the morphology of these neurons (somas more preserved than in the conventional immunostaining method and processes more delineated), although we did not find differences in the pattern of distribution of these cells. There were an abundant number of intense stained fibers without varicosities in a weakly stained neuropil as it was observed in sections processed by the conventional immunostaining method.

**Figure 5:**
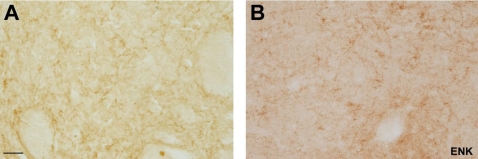
A, Immunohistochemistry for ENK in the caudate nucleus. Bright field photomicrographs in high magnification of ENK fibers without citraconic anhydride solution in the caudate nucleus. B, bright field photomicrographs in high magnification of ENK fibers with citraconic anhydride solution in the same nucleus. Note that the latter fibers are better delineated than in the conventional immunostaing method. Scale bar: 50 µm.

Finally, the immunostaining for ENK in the caudate nucleus in the formaldehyde fixed brains revealed numerous fibers with varicosities in a weakly stained neuropil, but no ENK-ir somata. These fibers were better delineated than in the conventional immunostaining method (Figure 5).

## Discussion

Fixation of tissue specimens in formaldehyde is a commonly used method in pathology laboratories and in brain banks, being the principal source of human brain samples for research studies (i.e. immunohistochemical studies in free-floating sections). However, this method based on fixation in formaldehyde for a long time does not allow an optimal localization and accessibility of antigens to their specific antibodies. Several AR methods have been developed to address this problem. The variety of methods undoubtedly contributes to discrepancies among the immunostaining results of different investigations [34].

In the present study, we have investigated a new AR method to optimize immunostaining in free-floating human brain sections, showing that, heating formalin-fixed tissue in 0.05% citraconic anhydre solution, pH 7.4, at 95°C for 45 minutes, restores the immunostaining for several antigens.

Temperature has been considered one of the most important variables for retrieval of antigens concealed by formalin fixation [4], [34]. Here, we have observed that a temperature of 95°C is optimal since it allowed us to retrieval antigens and does not damage the tissue.

In addition, different heating methods have been used for AR, such microwaving [4], autoclaving [6], [35], pressure cooking [36], steam heating [37] and water bath [7], [34]. In our study, the latter has been the one we have used because it allowed us to establish an optimal control of the samples.

Different solutions to AR combined with heat has been developed, all of then reviewed periodically [3]. The most commonly used has been citrate buffer (pH 6.0) and the Tris-HCl buffer containing 5% urea (pH 9.0) [34]. In a recent paper, Namimatsu et al. [34] have proposed a new AR method based on the use of citraconic anhydride, and they compared this protocol with citrate buffer and Tris-HCl in paraffin-embedded sections of different tissues, as tonsil, ovary, skin, lymph node, stomach, breast, colon, lung and thymus [34]. This group concluded that the AR method based on citraconic anhydride allowed them to carry out optimal immunostaining procedures in comparison of the existing AR protocols, so the extent and the intensity of immunostainings is enhanced [34]. Moreover, this new AR method provides some advantages, for instance the reliability, the possibility to establish as a universal AR method, and the possibility to process the slices in one batch [34].

Although Namimatsu et al. [34] have employed the citraconic anhydride in several tissues, as far as we know nobody has tested this AR method in human brain sections for immunohistochemical free-floating studies. When we did probes in human brain tissue, our results confirmed that employing the citraconic anhydride combined with heat as an AR method in human brain sections allowed us to carry out optimal free-floating immunohistochemistry procedures, independently of the fixation time in formaldehyde.

We have tested five antibodies commonly used in neurochemical human brain studies, specifically at subcortical levels (anteroventral thalamic nucleus, mediodorsal thalamic nucleus, substantia nigra and caudate nucleus). For example, the calcium-binding protein calbindin, which is an excellent neurochemical marker for the morphological study of the human thalamus and its nuclear subdivisions [15], [38]–[41] was successfully used in this study (Figure 1; see Figures 2–5 for immunostainings of SP, GAD, TH and ENK). Besides, it is interesting to note that the methodology we have described in this study has been successful in showing both projection neurons and interneurons in the human thalamus [42].

**Figure 6:**
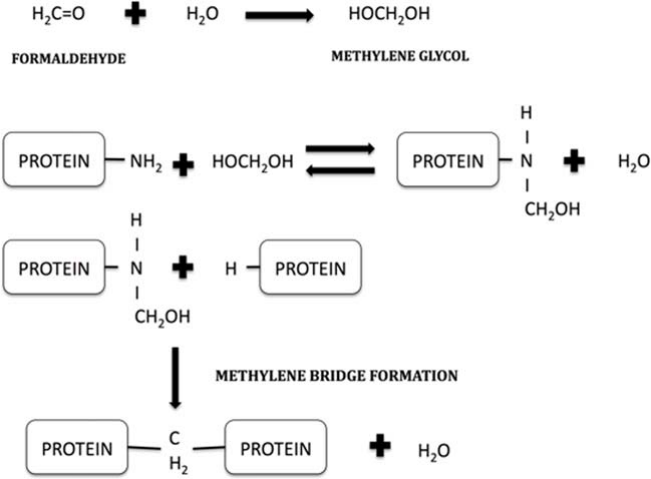
Schematic view of formaldehyde fixation stages. On the top: formation of methylene glycol by the addition of water and formaldehyde. On the bottom: formaldehyde reacts with various parts of proteins molecules forming adducts. This reaction is followed by the formation of methylene bridges between closed proteins.

The samples used in the present study had variable fixation times, ranging from 10 days to 7 years (Table 1). As we said, it is a fact well established that formaldehyde fixation compromises immunoreactivity of some antigen, not usually by damaging or removing the antigens but by preventing contact between epitopes and antibodies. The way that tissue molecules are modified affects the outcome of immunohistochemical staining [3]. Basically the fixation process has three stages. Firstly, the formaldehyde dissolves in water, combining with it to form methylene hydrate (methylene glycol). Then, it can react with almost all end groups found in biological molecules such as amines, amides, hydroxyls, sufhydryls, reactive hydrogen atoms from aromatic amino acids, etc; the aldehyde combination result in the attachment of various hydroximethyl adducts at the original end groups [43]. Finally, this addition is followed by crosslinking with other end groups by the formation of a methylene bridge -CH_2_- (Figure 6) [3].

The particular reaction of formaldehyde with proteins has been studied extensively. The aldehyde group can react with some atoms of proteins like nitrogen, oxygen, sulphur or carbon, etc. [44]. A well-studied example of this reaction is the crosslink between the nitrogen atom at the end of the side chain of lysine and the nitrogen atom of a peptide linkage (Figure 6).

The fixation by formaldehyde is almost completed in 24 hours but the formation of methylene bridges proceeds much more slowly [45]. Formaldehyde also affects nucleic acids by forming adducts with exocyclic nitrogen atoms in nucleotides [46]. This immobilization of DNA and RNA is also attributed to trapping of the long nucleic acid molecules in networks of associated basic protein molecules, which are cross-linked by methylene bridges. Other substances such as carbohydrates, lipids, and so on, are trapped in a matrix of insolubilized and crosslinked protein molecules but are not chemically changed by formaldehyde unless fixation is prolonged for several weeks [44].

In summary, we have reported a novel method to carry out immunohistochemical studies in free-floating human brain sections. Because fixation of brain tissue specimens in formaldehyde is a commonly method used in brain banks, this new AR method could facilitate immunohistochemical studies of brains with prolonged formalin fixation.
